# Mechano-signaling in heart failure

**DOI:** 10.1007/s00424-014-1468-4

**Published:** 2014-02-16

**Authors:** Byambajav Buyandelger, Catherine Mansfield, Ralph Knöll

**Affiliations:** Imperial College, British Heart Foundation—Centre for Research Excellence, National Heart and Lung Institute, Imperial Centre for Translational and Experimental Medicine, Hammersmith Campus, Du Cane Road, London, W12 0NN UK

**Keywords:** Mechanosensation, Mechanotransduction, Mechanoelectric feedback, Electromechanic feedback, Heart failure

## Abstract

Mechanosensation and mechanotransduction are fundamental aspects of biology, but the link between physical stimuli and biological responses remains not well understood. The perception of mechanical stimuli, their conversion into biochemical signals, and the transmission of these signals are particularly important for dynamic organs such as the heart. Various concepts have been introduced to explain mechanosensation at the molecular level, including effects on signalosomes, tensegrity, or direct activation (or inactivation) of enzymes. Striated muscles, including cardiac myocytes, differ from other cells in that they contain sarcomeres which are essential for the generation of forces and which play additional roles in mechanosensation. The majority of cardiomyopathy causing candidate genes encode structural proteins among which titin probably is the most important one. Due to its elastic elements, titin is a length sensor and also plays a role as a tension sensor (i.e., stress sensation). The recent discovery of titin mutations being a major cause of dilated cardiomyopathy (DCM) also underpins the importance of mechanosensation and mechanotransduction in the pathogenesis of heart failure. Here, we focus on sarcomere-related mechanisms, discuss recent findings, and provide a link to cardiomyopathy and associated heart failure.

## Introduction

Mechanosensation is a fundamental process in biology [23] and particularly important for the heart [17]. However, mechanosensation is a broadly applied term with no clear definition in the biological sciences. There are different types of mechanical stimuli and it is important to differentiate between them. Stress (*σ*) (dimension: newton per square meter) is physically defined by:

$$ \sigma =\frac{F}{A} $$

where *F* is the applied force per unit area (*A*). Also, “shear stress” (*τ*) (dimension: newton per square meter), where the applied force (*F*(*S*) = shear force) acts parallel to the area (*A*), is shown as:

$$ \tau =\frac{F(S)}{A} $$

Other types of physical stresses such as compression and torsion may also occur and are equally important. Distinct from stress is “strain” (*ε*) (dimensionless) which is physically defined by:

$$ \varepsilon =\frac{\varDelta L}{L_0} $$

where *L*_0_ is the initial length and Δ*L* is the change in length. Another important parameter is Young’s modulus (dimension: newton per square meter), which is a measure of the stiffness:

$$ E=\frac{\sigma }{\varepsilon } $$

Cells are able to detect stress and strain via changes in conformation of proteins or macromolecular protein complexes, but the precise molecular mechanisms remain unclear. In this regard, two different models have been developed to explain mechanosensory behavior (Table 1): (1) the localized and (2) the decentralized model. The localized model proposes that changes at the cell membrane are sensed immediately and are transmitted from there to other parts of the cell. In contrast, the decentralized model proposes that any force applied at the cell surface will cause deformations of elastic cytoskeletal components and, as such, can be sensed far away from the area of impact. The latter model is also called the “tensegrity” model (derived from tensional integrity) based on Buckminster Fuller’s geodesic dome.

**Table 1:** Summary of the localized and decentralized models of cardiac mechanosensation

Localized mechanosensation	Decentralized mechanosensation (tensegrity)
Mechanosensitive ion channels	Sarcomere related signaling:
Transmembrane receptors	Z-disc titin/MLP/telethonin
Integrins/dystrophin	Titin kinase/MURF1 and 2/nbr1/p62
Angiotensin receptor	I-band titin/FHL1/ERK/MARP
Caveolae	Actomyosin interaction
	Protein-protein interaction-mediated processes:
	Kinases (MAPK)
	Phosphatases (calcineurin)
	Other posttranslational modifications

The concept of tensegrity has profound consequences for cell biology as it implies that nearly every protein is involved in mechanosensation and mechanotransduction. Therefore, naturally occurring mutations in these components or complete loss of these components such as those observed in mouse knockout studies are all expected to affect mechanosensory functions.

Different mechanosensory protein complexes can be found in cardiac myocytes: (1) cell membrane associated, (2) intracellular embedded, and (3) sarcomere related (Table 1). All these various signalosomes are sensitive to different types of mechanical stimuli. For example, a deformation of the cell membrane may be detected by cell membrane-associated signalosomes, such as stretch-activated channels, angiotensin receptors, the caveolae, and integrin-mediated signaling. Depending on severity and duration, these events may also be sensed by intermediate filaments and/or by sarcomere-associated signalosomes. Here, we focus on mechanical stimuli and try to put them into a physiological perspective (please see [29] for a recent review on hormonal stimulators of hypertrophy).

## Heart failure, hypertrophy, and mechanical forces

Heart failure is the leading cause of death worldwide [26]. While we continue to unravel the genetic basis of heart failure and while the equilibrium between cardiomyocyte loss and regeneration is severely damaged in heart failure, the functional link between both and how mechanical forces influence these events remain not well understood [33]. This fact is underscored by the recent discovery that the frequency of potential heart failure causing mutations is much higher than the prevalence of heart failure itself [14]. It might well be that the magnitude of mechanical forces combined with genetic defects in mechanosensation or mechanotransduction triggers pathological effects and disease. Cardiac hypertrophy (or remodeling [19]) and its reverse, cardiac atrophy, are triggered, among others, by an increase or decrease in physical stress, respectively. At the organ level, these changes lead to significant remodeling processes, including changes in angiogenesis and the composition of the extracellular matrix [19].

At the cellular level, cardiac hypertrophy and atrophy are associated with an increase or decrease in cell size, respectively, which alone poses a tremendous challenge for every cell. These changes are particularly important for cardiac myocytes not only because new sarcomeres have to be added in parallel (during pressure overload) or in series (during volume overload) or they have to be removed in atrophy which can be described as remodeling in three dimensions. However, membrane constituents have to increase or decrease proportionately which can be described as remodeling in two dimensions. Due to transcription factors being unable to separately regulate single genes (i.e., there are no specific sets of transcription factors available to separately regulate the transcription of membrane or cellular components), membrane and cellular components have to change proportionately and a new equilibrium has to be found, which is only possible within certain limits. Therefore, it is no surprise that lethality after myocardial infarction is highest immediately in the days after the incident where remodeling occurs [44]. Interestingly, these arrhythmias can be efficiently terminated by overexpression of a dominant negative mechanosensitive KCNH2 channel (KCNH2-G628S) or by application of the KCNH2 channel blocker dofetilide [1]. This result is interesting because ischemia/reperfusion is associated with changes in the expression of a multitude of mechanosensitive channels such as TRPC6 [24] (please see for a brief review: [47]). Moreover, stretch is known to have a preconditioning effect on ischemia/reperfusion [39] which is sensitive to the inhibition of K_ATP_ channels [36]; therefore, interference with KCNH2 could have a negative impact on cell survival. TRPC6 is regulated by the calcineurin/nuclear factor of activated T cells (NFAT) pathway [24] and negatively regulates endothelin which leads to antifibrotic effects—a hallmark of physiological hypertrophy [29]. Also, overexpression of calcineurin has protective effects in a murine model of dilated cardiomyopathy (DCM) [13]. While calcineurin/NFAT signaling is largely seen as an inductor of pathological hypertrophy, this pathway can also play a role in physiological hypertrophy.

Therefore, we argue that no signal transduction pathway is adaptive or maladaptive, rather the strength of the stimulus, its nature (i.e., persistent such as in aortic constriction or intermittent such as during exercise), and the genetic composition of the organism will decide if the stimulus results in heart failure. Although models have been reported whereby hypertrophy was not necessary to sustain elevated biomechanical stress [7], hypertrophy, within limits and if reversible, is initially beneficial. From a signaling point of view, hypertrophy is expected to prolong, while atrophy is expected to shorten signal transduction from the cell membrane to the nucleus.

## Sarcomere-related mechanisms of mechanosensation

**Fig. 1:**
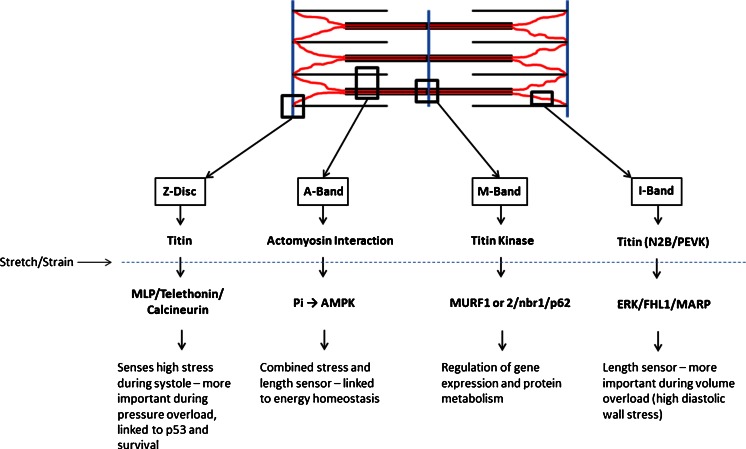
Schematic representation of sarcomere-associated mechanosensory processes

In contrast to other cell types, striated muscle cells are unique in that their primary function is to generate forces which enable the circulation of blood or movement of the body. These forces need to be “fine-tuned” and carefully controlled which may be defined as “sensing of the magnitude and dynamics of contractility,” as opposed to the well-known concepts of the “perception of extracellular mechanical stimuli”—a general description of mechanosensation which can be found in every living cell [22]. From this point of view, it is therefore not surprising that several mechanosensitive signalosomes are present in sarcomeres (Fig. 1).

During the course of the last decade, much attention has been focused on the passive elastic components of the sarcomere as mechanosensors, particularly the giant proteins titin, nebulin (nebulette in the heart), and obscurin [29]. However, Z-disc-related mechanosensation remains not well understood. The Z-disc is made up of a lattice of α-actinin molecules, which anchor the ends of actin filaments, and thus transmits force from the molecular motor to adjacent sarcomeres and ultimately to the ends of the contractile cell. Apart from transmitting forces to the ends of the cell, each Z-disc is also connected to the extracellular matrix (ECM). Hence, force transmission to the cavity of the heart also, and maybe predominantly, occurs via the Z-disc-integrin-ECM mechanical pathway. The Z-disc is flexible and muscle shortening, which causes high systolic wall stress, changes the conformation of the *α*-actinin lattice. This may enable sensation of tension (*σ* = *F*/*A*), which may involve a signalosome consisting of muscle LIM protein (MLP, CSRP3), telethonin (TCAP), and the amino-terminal titin [18]. Loss of MLP in genetically altered animals or MLP mutations in humans are associated with various types of cardiomyopathy and associated heart failure. For example, the 10 T → C (Trp4Arg) MLP missense mutation has been identified in several cardiomyopathy patients and is present at high frequencies (up to 1 %) in some European populations [20], and the corresponding knock-in animals develop an age and gene dosage-dependent hypertrophic cardiomyopathy and heart failure phenotype. At the molecular level, this mutation is associated with significantly decreased MLP mRNA and protein levels. Loss of MLP has also been reported in heart failure [50], and loss of MLP-mediated signaling may contribute to the development of this condition. Furthermore, W4R-MLP is associated with loss of affinity to its binding partner telethonin and translocates from the sarcomeric Z-disc to the nucleus (for more detailed reviews on MLP, please see: [6, 10]).

Telethonin (also known as titin-cap or t-cap) is a 19-kDa Z-disc protein with a unique β-sheet structure which assembles in a palindromic way with the N-terminal portion of titin [51] and thereby may constitute a signalosome participating in the process of cardio-mechanosensing. A variety of telethonin mutations are associated with the development of congenital diseases, including limb girdle muscular dystrophy 2G [8, 35, 38], hypertrophic cardiomyopathy (HCM), DCM [4, 12, 18], and intestinal pseudo-obstruction [32].

Telethonin-deficient and transgenic animals were generated, and it was shown that this protein is not an indispensable component of the titin-anchoring system, nor is deletion of the gene or cardiac-specific overexpression associated with a spontaneous cardiac phenotype, at least in mouse. It was found that a main novel function of telethonin is to modulate the turnover of the proapoptotic tumor suppressor p53 after biomechanical stress in the nuclear compartment, thus linking telethonin directly to apoptosis—a process which may be called mechanoptosis (i.e., apoptosis after biomechanical stress or a mechanosensitive type of cell death). In addition, loss of telethonin mRNA and nuclear accumulation of this protein is associated with human heart failure, an effect which may contribute to enhanced rates of apoptosis found in these hearts [21]. Mechanoptosis may be defined as a new type of apoptosis found only in striated muscle which is (1) induced via increased mechanical stress and (2) modified by muscle-specific proteins such as telethonin. However, it remains to be analyzed whether telethonin also affects other types of cell death such as necroptosis or processes such as autophagy.

These data support the concept of using antiapoptotic strategies to avoid the loss of cardiac myocytes [11]; however, using growth factor treatment as an antiapoptotic strategy in heart failure patients was not successful which possibly highlights our lack of knowledge of the underlying molecular mechanisms and hence our inability to interfere [15].

In addition to the chronic adaptive mechanisms described above, which are linked to gene expression, stretch also causes a more immediate change in actomyosin cross-bridge kinetics. It was recently shown that stretch applied to activated cardiac trabeculae is related on a millisecond timescale to a rise in force and to a decrease in Pi production and, therefore, decreased ATP hydrolysis, the latter lasting for the whole duration of stretch [2, 30]. This property seems to be intrinsic to the cardiac contractile apparatus and indicates an increase in the force economy of the cross-bridges after stretch. The change in the rate of Pi release may also affect homeostasis of cellular energy metabolism through Pi feedback pathways [45], which may be sensed by the adenosine monophosphate kinase (AMPK), an enzyme already implicated in mechanosensation—at least in epithelial cells [48]. In fact, AMPK is localized to the nucleus and cytoplasm [43] and, therefore, may well be able to sense changes in sarcomeric AMP/ATP ratios. Moreover, mutations in this gene are a well-known cause of HCM [3], and AMPK activity is necessary for stress-related survival of many cell types, including tumor cells [16]. Cardiomyopathies such as HCM are often associated with increased force production, but decreased efficiency. AMPK may well be able to sense these changes and translate them into long-term effects such as hypertrophy and/or cell survival-related pathways. It would be interesting to analyze these effects in heart failure, where βMHC is upregulated and which is known to have slower ATPase activity.

## N2A and N2B—titin mechanosensor complexes

With a molecular mass of up to 4.2 MDa, titin is the largest molecule in biology and well known for its multiple functions such as serving as a molecular ruler, its importance during embryonic development, and for its role in providing mechanical stability. Two isoforms of titin are found in the adult heart: the N2B and N2BA isoforms. Both isoforms contain N2B, PEVK, and multiple immunoglobulin (Ig) domains, with the N2BA isoform also containing an additional N2A domain. All of these domains unfold upon stretch and/or store energy during every cycle of contraction and relaxation via entropic springs [28].

Smaller mammals such as the rat and mouse express mainly the shorter and stiffer N2B isoform with larger mammals expressing both the N2B and the longer and more compliant N2BA isoform. During postnatal development, stiffness increases due to switching of fetal cardiac titin (a more compliant isoform) to adult N2B and N2BA isoforms. Diseases such as DCM have also been shown to affect the N2BA/N2B ratio to varying extents. An increase in titin-mediated stiffness has been observed in a tachycardia-induced DCM canine model [49], while a decrease in titin stiffness has been seen in patients with end-stage DCM, whereas an increase has been seen in patients with earlier stage disease [27]. It remains to be elucidated whether different types of pathological stimuli, species differences, early versus late effects of disease, or different diseases per se specifically modify titin-mediated stiffness.

The N2B domain binds specifically to four and a half LIM protein 1 (FHL1), which in turn is the core of a signalosome consisting of RAF, MEK1/2, and ERK2, thus connecting growth factor-mediated Gq signaling and extracellular signals to titin extensibility and finally to changes in gene expression. Interestingly, loss of FHL1 blunts pathologic hypertrophy, and as such, inhibition of this pathway might be beneficial [46].

Another important pathway is linked to the N2A elastic titin domain, where the muscle ankyrin repeat proteins (MARP) including cardiac ankyrin repeat protein (CARP), ankrd2/Arpp, and DARP interact to constitute a signalosome which responds to passive stretch in vitro [34].

## Titin kinase mechanosensor complex

While titin’s elastic I-band domains may be able to primarily sense strain and titin’s Z-disc anchored amino-terminus is involved in the sensation of stress, titin also contains a kinase domain which is involved in mechanosensation. Interestingly, titin’s M-line domain contains a mechanically modulated kinase able to bind and phosphorylate nbr1 and p62 in vitro. MURF1 and 2 (and probably MURF3 which has not been analyzed yet) also bind to this complex and will translocate into the nucleus upon mechanical inactivity, where they downregulate and/or induce the nuclear export of serum response factor (SRF) and, as such, aggravate the transcriptional atrophy program [5]. This is supported by the R279W-titin kinase mutation which is associated with hereditary myopathy with early respiratory failure (HMERF) and which leads to a dramatic loss of affinity to nbr1 [25]. Additional evidence for this model is supported by in vitro experiments whereby stretching of the kinase domain leads to activation of the kinase, thus effectively linking mechanosensation to kinase activity (“mechanozymatics”) [42]. Moreover, titin’s kinase domain is linked via nbr1 and p62 to autophagy, a process of regulated protein and organelle turnover [9].

However, the mechanism of tail release of the titin kinase remains unclear [31], HMERF patients develop primarily a skeletal muscle phenotype, and recently, mutations outside the titin kinase domain have been found to cause HMERF, which points to the presence of additional mechanisms [37, 41]. Clearly, inducible pluripotent stem cell-derived systems and essential gain and loss of function models are needed to understand the titin kinase-mediated pathways better.

## Summary

Titin’s N2A, N2B, and titin kinase domains may primarily function as length sensors (i.e., *ε* = ∆*L*/*L*_*0*_) during diastole where wall stresses are relatively low. This function is important during volume overload, which leads to the addition of sarcomeres in series. In contrast, the sarcomeric Z-disc may function as a stress sensor (*σ* = *F*/*A*) particularly during systole where wall stresses are high, where sarcomeres are added in parallel, and where cell survival pathways are activated to avoid apoptosis. Other stresses such as shear stress and torque may also be sensed by the Z-disc. The known macrostructural changes of the sarcomeric Z-disc from the “basketweave” formation in systole to the “small lattice” formation in diastole may play a role here, but these conformational changes remain poorly understood [40]. Aside from that, actomyosin interaction may also link stretch to energy homeostasis (Fig. 1).
